# Rediscover Neighborhood: A Synthesis of Lived Experiences Following Dementia Diagnosis Through Walking Interview

**DOI:** 10.1093/geroni/igaf122.1243

**Published:** 2025-12-31

**Authors:** Wenjin Wang, Ziying Zhang, Xi Chen, Chanam Lee

**Affiliations:** Texas A&M University, College Station, Texas, United States; Simon Fraser University, Burnaby, British Columbia, Canada; Texas A&M University, College Station, Texas, United States; Texas A&M University, College Station, Texas, United States

## Abstract

Around 85% of older adults with dementia in the US reside within the community (NHATS, 2019). However, many do not venture far from their homes due to fears of getting lost or the loss of their driving ability. Therefore, the immediate neighborhood environment plays a crucial role in supporting their independence. Walking interview is an effective in-situ, participatory method to capture the lived experiences and environmental interactions of persons with dementia (PwDs), yet studies on walking interviews with PwDs is often limited by small sample sizes and a narrow focus. A systematic review and meta-synthesis can bridge these gaps and offer a critical, comprehensive view of neighborhood experiences after dementia diagnoses. We included 17 papers that involved 172 PwDs from four countries. Guided by the Contexts for Development and Aging (CODA) framework, we found that environmental features (e.g., street crossings, sidewalks, and landmarks) can either support or challenge PwDs’ outdoor mobility, yet many adapt by developing unique strategies, modifying daily routines, or relying on others. From the social perspective, PwDs form a vital, reciprocal network with family, friends, neighbors, and even familiar “strangers”. Technology further expands PwDs’ physical and social experiences by facilitating location tracking and virtual interactions, despite potential accessibility barriers and misuse. The study revealed how PwDs address vulnerability and adapt with resilience in neighborhoods as dementia progresses. More research is needed on the neighborhood experiences of PwDs, focusing on translating their daily realities into actionable frameworks for dementia-friendly communities.

